# Effects of eggshell and seashell powder as natural dietary calcium supplements on growth, molting frequency, and carapace calcium composition of juvenile red claw crayfish, *Cherax quadricarinatus*

**DOI:** 10.7717/peerj.15449

**Published:** 2023-07-12

**Authors:** Ahmad Shahroom, Rossita Shapawi, Saleem Mustafa, Nur Fatihah Abd Halid, Abentin Estim, Audrey Daning Tuzan

**Affiliations:** Borneo Marine Research Institute, Universiti Malaysia Sabah, Kota Kinabalu, Sabah, Malaysia

**Keywords:** Red claw crayfish, Calcium supplement, Natural calcium source, Growth performance, Molting

## Abstract

The growth performance of red claw crayfish, *Cherax quadricarinatus*, fed diets with different sources and concentrations of natural calcium was evaluated. Formulated diets containing 30% protein and 8% lipid were prepared using supplementation of 0 (control), 3%, 6%, 9% eggshell, and 6% seashell. *C. quadricarinatus* juveniles with an average total weight of 0.21 ± 0.01 g and length of 25.0 ± 0.10 mm were obtained from ten ovigerous females bred in captivity. An aquarium with a size of 0.72 m² was divided into eight compartments with an area of 0.09 m² per compartment and was individually stocked with juvenile crayfish. A total of eight juvenile crayfish were used per dietary treatment. There were five dietary treatments in total and each of these treatments were replicated three times. The addition of eggshell and seashell powder exerted positive effects on the growth performance, molting frequency, and survival of the crayfish. Crayfish fed with a 6% eggshell-supplemented diet exhibited the best overall growth performance. The calcium percentage of the carapace increased with the increase in calcium content of the diets. Meanwhile, the performance of diets comprising eggshells and seashells was not significantly different (*P* > 0.05). Considering the low cost and abundance of eggshells, the addition of 6% of this product to the diet is recommended for the best growth of juvenile *C. quadricarinatus*.

## Introduction

Australian red claw crayfish, *Cherax quadricarinatus*, locally known as freshwater lobster, is a crustacean with several excellent attributes as an aquaculture species. Even though crayfish aquaculture has been established now for more than 25 years, the total production is still quite small and largely based on best management practices (Fisheries & Aquaculture Department, 2011). Crayfish’s potential as an aquaculture species is based on its flexible feeding habits, fast growth rate, and tolerance to different environmental conditions. In Malaysia, the aquaculture of crayfish started in the early 2000s (Alimon et al., 2003). There is a growing interest in its farming due to the lucrative price of RM100–RM150 (= US$23–34.5) per kg in the Malaysian market. Nevertheless, crayfish aquaculture in Malaysia is still in its infancy and farmers are trying to follow the methods established in Australia such as semi-intensive batch production in the earthen pond (Curtis & Jones, 1995) with some local adaptations.

In any aquaculture enterprise, cost-effective feed is one of the key factors determining its economic viability. For the crayfish, no specific feed is commercially available in Malaysia. Local farmers are mostly using raw foods, especially mostly vegetable-based diets or a variety of low-cost products which are not prepared to meet the nutritional requirements of crayfish, resulting in their sub-optimal growth performance. In fact, high mortality was observed during the molting phase or known as molt death syndrome (MDS) (Bowser & Rosemark, 1981), obviously due to the poor state of nutrition and general well-being of the crayfish. Mineral supplementation of feeds is considered a cost-effective solution. Several types of minerals such as calcium and magnesium improve immunity, health, muscle development, enzyme systems, and production efficiency in aquaculture (Boggino, 2022). Despite their importance, there are glaring knowledge gaps in the mineral requirements of crustaceans especially, crayfish. Several attempts have been made (NRC, 2011; Shiau & Hsieh, 2001; Cheng et al., 2005; Truong et al., 2020), but even for shrimps, which have been far more thoroughly investigated, requirements of micro-and macro-minerals are still not well understood (NRC, 2011). To formulate a balanced diet, all macro- and micro-nutrient requirements must be known. Organic minerals from eggshells and seashells have greater bioavailability in aquatic animals (Towers, 2016). This implies their better absorption and utilization which are good for growth and less discharge that reduces the environmental impact. Crayfish can absorb calcium directly from food and water (Lovell, 1989) and old shells to make a reserve in the body (Pavey & Fielder, 1990). Riza (2009) also explained that supplementing calcium directly to the feed is much more efficient due to calcium being digested directly with the feed. The use of eggshells and seashells can produce multiple advantages in crayfish aquaculture by supplying sufficient calcium which is needed for normal growth, molting, and other physiological functions (Lall, 2002). The composition of eggshells is approximately 98.2% calcium carbonate, 0.9% magnesium, and 0.9% phosphorous (phosphate) (Shwetha et al., 2018) and according to Nys & Gautron (2007), eggshell is made up of calcium carbonate (95%) and a minor amount of organic matrix (5%). Rao et al. (2016) stated that seashells are excreted from animal mantle which is made up of mostly calcium carbonate. Seashell consists of 95% calcium carbonate and the remainder are organic matter and other compounds (Hamester, Balzer & Becker, 2012).

Although no comprehensive scientific study has so far been made on the use of these by-products as sources of natural calcium in crayfish farming, there are studies utilizing calcium carbonate powder as a calcium source in feed (Mazlum & Şİrİn, 2020), suggesting a recommendation level of 6%. Egg shells as well as shells of bivalves are discarded products available in most countries. Their recycling and reusing in aquaculture contribute to the environmental compatibility of this sector (Hargreaves, 2020; Colombo & Turchini, 2021). Aquaculture’s growing contribution to global food security requires a new focus on exploring raw materials that are wasted and valorizing them (Fraga et al., 2022). A survey of published information on the adoption of circular economy in aquafeed production carried out by Kusumowardani & Tjahjono (2020) emphasizes that far more efforts are needed toward translating the circularity models for aquafeed into commercial aquaculture.

The present investigation was carried out to determine the effects of eggshell and sea-shell powder as natural calcium supplements in diets. The incorporation of locally available ingredients from discarded products in aquafeed will lower the feed price and consequently reduce the cost of crayfish production. Therefore, more research is necessary to understand crayfish calcium uptake. This study also serves to demonstrate the relevance of applying circular economy principles in sustainable aquaculture.

## Materials and Methods

### Ethics statement

All the animal experiments were performed according to University Malaysia Sabah (UMS) rules and regulations in animals for the Care and Use of Animals for Scientific Purposes and complied with the European Union Directive (2010/63/EU).

### Experimental crayfish

A total of 10 ovigerous female red claw crayfish with an average total length of about 100 mm were collected from a local breeder at Beringgis, Papar, Sabah. They were transported to Sabah Green Sanctuary Hatchery where spawning took place. The crayfish were kept in square-shaped fiberglass tanks provided with shelter (polyvinyl chloride pipe). Recirculating aquaculture system (RAS) was used with an inbuilt filtration mechanism. The female was fed with commercial freshwater prawn feed (Dindings, Kuala Lumpur Malaysia; crude protein 30%, lipid 5%). Hatched larvae were reared for 1 month. The total weight (TW) of subsamples was measured to the nearest milligram to estimate the size of the initial crayfish.

### Culture conditions

The experiment was conducted using an individual culture system with a total of 120 juvenile crayfish allocated into 15 aquaria with a surface area of 0.72 m $^2$ (120 cm long, 60 cm width, 14 cm height) filled with 100 L of filtered tap water in a closed system. Each aquarium was divided into eight compartments with an area of 0.09 m² per compartment and was stocked with one juvenile crayfish per compartment. A total of eight juvenile specimens were used per dietary treatment. There were five dietary treatments in total and each of the treatments were replicated three times. The aquaria were placed in a room with natural light (8 h day light time) and supplied with continuous aeration. RAS was used for this experiment. Water quality was monitored every week except for temperature which was measured twice daily at 8.00 am and 8.00 pm using a thermometer. The water temperature inside the tanks ranged from 25.0–27.0 °C, while pH varied from 7.0–7.9 (Waterproof pHep®5 pH/Temperature Tester, Model HI98128, Hanna Instruments, Woonsocket, RI, USA) during the experiment. Ammonia nitrogen N-NH4 and nitrite nitrogen N-NO2 were determined using API Freshwater Master Test Kit.

Specimens of juvenile crayfish with an average total length of 25.0 ± 0.10 mm (TL) and weight of 0.21 ± 0.01 g were randomly distributed in each aquarium and acclimated to the experimental condition for 5 days before the commencement of feeding. All the crayfish in each aquarium were weighed at the end of the experiment. The aquarium was provided with a small section of polyvinyl chloride (PVC) pipe (diameter 1.25 cm, length 10 cm) to serve as a shelter.

### Experimental diets and feeding trial

Five experimental diets were formulated (30% crude protein and 8% crude lipid) to provide four levels of calcium supplementation using control (0% ES), 3% (3% ES), 6% (6% ES), and 9% eggshells (9% ES), and one level of calcium supplementation using 6% seashell (6% SS). To compare the performance of these two natural calcium sources, only a 6% inclusion level was selected for seashells based on the previous study (Mazlum & Şİrİn, 2020). Eggshells and seashells are abbreviated as ES and SS, respectively. Fermented soybean meal and palm oil were used as dietary protein and lipid sources, respectively. Samples of eggshells were collected from the eggs of domestic fowl (*Gallus gallus*) that are widely consumed. The adhering membranes were removed, and the shells were washed. Discarded shells of mixed bivalves were washed with potable water. Subsequently, the eggshells and seashells were air dried and placed in an oven maintained at 100 °C until a constant weight was attained, generally in about 10 h.

Eggshells and seashells were then blended into a fine powder using a kitchen blender and mixed homogeneously. All dry ingredients were mixed in a mixer and water (40% of the diet) was added to the mixes to form a soft dough. The dough was passed through a mincer with a 2-mm-diameter pellet die. The pellets were air-dried at 60 °C in an oven and stored at 5 °C until use. Crayfish were fed with experimental diets to apparent satiation (*ad libitum*) twice a day at 3:00 pm and 10:00 pm for 60 days. Uneaten feed was siphoned out daily on a regular basis in the morning at 8:00 am. The formulation and proximate composition of the diets are shown in Table 1.

**Table 1:** Ingredient and proximate composition of experimental diets (% dry matter basis).

	Calcium source/Calcium percentage				
Ingredients	0% ES	3% ES	6% ES	6% SS	9% ES
^a^Soybean meal	61.5	61.5	61.5	61.5	61.5
^b^Corn starch	25	22	19	19	16
^c^CMC	1.5	1.5	1.5	1.5	1.5
^d^Vitamin premix	3	3	3	3	3
^e^Mineral premix	2	2	2	2	2
^f^Palm oil	7	7	7	7	7
^g^Eggshell	0	3	6	0	9
^h^Seashell	0	0	0	6	0
Proximate composition					
Moisture	12.2	12.4	12.3	12.4	12.2
Crude protein	30.3	30.1	30.3	30.4	30.2
Lipid	8.1	8.1	7.9	8.2	7.8
Ash	10.8	11.2	11.3	10.7	10.5

**Notes:**

a GENTIDE fermented soybean, China.

b STAR brand. Giant Sdn. Bhd.

c Carboxymethyl cellulose (CMC), Sigma.

d Vitamin mixture (g/kg mixture): Ascorbic acid, 45.0; Inositol, 8.0; Choline chloride, 75.0; Niacin, 4.5; Riboflavin, 1.0; Pyridoxine HCL, 1.0; Thiamine HCL, 0.92Retinyl acetate, 0.60; Vitamin D3, 0.083; Menadione, 1.67; DL alpha tocopherol acetate, 8.0; D-biotin, 0.02; Folic acid, 0.09; Vitamin B12, 0.00135.

e Mineral mixture (g/kg mixture): 327.0; Ferrous sulphate, 25.0; Magnesium sulphate, 132.0; Potassium chloride, 50.0; Sodium chloride, 60.0; Potassium iodide, 0.15; Copper sulphate, 0.785; Manganese oxide, 0.8; Cobalt carbonate, 1.0; Zinc oxide, 3.0; Sodium salenite, 0.011.

f Sri Murni Brand Palm Oil.

g Eggshells collected from local restaurants, Restaurant Jamal Saleem.

h Seashells collected from the local beach, Tanjung Aru Beach.

### Sample collection and analysis

The number of surviving juveniles and their body weight were determined at the end of the feeding trial. Juveniles were placed on a filter paper to remove excess water and weighed to the nearest 0.01 g. Weight gain (%), specific growth rate (SGR), feed conversion ratio (FCR) survival, molting frequency were calculated using Eqs. (1)–(5):

(1) $${\rm{Weight}}\;{\rm{gain}}{\mkern 1mu} (\% )\,{\rm{ = }}\,[{\rm{Final}}\;{\rm{weight}}\; - \;{\rm{Initial}}\;{\rm{weight}}]{\rm{/}}[{\rm{Initial}}\;{\rm{weight}}] \times {\rm{100}}$$

(2) $${\rm Specific\; growth \;rate\, (SGR, \%/d) = [(ln \;final\; weight - ln\; initial\; weight) / days] \times 100}$$

(3) $${\rm Feed \;conversion \;ratio \;(FCR) = feed \;fed \;(g) / weight\; gained \;(g) }$$

(4) $${\rm Survival \;rate \;(SR, \%) = (final\; number\; of \;crayfish/initial \;number \;of\; crayfish) \times 100}$$

(5) $${\rm Molting \;frequency = Total\; number\; of\; molting/number \;of \;crayfish}$$

### Calcium and phosphorus analysis

Calcium and phosphorus content in the eggshells, seashells, experimental diets, and exoskeleton were determined using an Inductively Coupled Plasma—Optical Emission Spectroscopy (ICP-OES OPTIMA 8000). The test method was based on AOAC 999.11-2016 (Maage, 2013). On completion of the feeding trial, the exoskeleton was removed from the crayfish body for calcium analysis. The calcium content in the exoskeleton was also determined by using the same equipment analysis.

### Water quality

During the experiment, a pH range of 7.7–7.9, ammonia range of 0.17–0.21 mg/L, nitrite range of 0.30–0.36 mg/L, and general water hardness range of 210–230 mg/L were observed. Throughout the experiment, the nitrite level was maintained below 0.4 mg/L. The ammonia level during the experiment was maintained below 0.5 mg/L to prevent stress. The temperature of the water during the same period varied from 26.0 °C to 27.0 °C as shown in Table 2

**Table 2:** Water quality parameters (mean ± SD) during the feeding trials.

Water parameter	Recirculating Aquaculture System (RAS)		
	Replicate 1	Replicate 2	Replicate 3
Temperature (°C)	26.20 ± 0.70	26.50 ± 0.30	26.38 ± 0.69
pH	7.70 ± 0.16	7.81 ± 0.07	7.83 ± 0.03
Total ammonia (mg/L)	0.16 ± 0.01	0.20 ± 0.03	0.15 ± 0.01
Nitrite (mg/L)	0.31 ± 0.01	0.34 ± 0.01	0.30 ± 0.01
Hardness (mg/L)	218.54 ± 3.47	225.24 ± 2.64	212.46 ± 1.28

### Statistical analysis

The data on weight gain, SGR, feed intake, FCR, survival rate, molting frequency, and calcium content in the exoskeleton were analyzed using variance analysis (one-way ANOVA) to identify the significant difference among treatments. Levene’s test and Tukey’s multiple range test were conducted to identify homogeneity of variance and multiple comparisons among treatments respectively. In the statistical analysis, differences were regarded as significant if the level of probability was less than 0.05 (*P* < 0.05). Arcsine was used to transform data in percentage before further analysis. R-test for correlation was conducted to identify the relationship between selected growth performance variables, molting frequency with body weight gain, and calcium content in carapace with the survival rate of juvenile crayfish. Values were presented as mean ± standard deviation (SD). All statistical analysis were carried out using statistical package IBM SPSS Statistics 20 for Windows.

## Results

### Calcium and phosphorus compositions

Eggshells consist of 34.40% calcium and 0.27% phosphorus while seashells consist of 38.04% and 0.03% calcium and phosphorous, respectively (Table 3). There are significant differences in the calcium-phosphorus ratio between experimental diets (one-way ANOVA, F = 29142.75; dF = 4.10; *P* = 0.00) except for diets 6% SS and 9% ES. Both 6% SS and 9% ES diets have calcium-phosphorus ratios of 3.06 and 3.04, respectively (Table 4).

**Table 3:** Percent composition of calcium and phosphorus in seashells and eggshells.

	Calcium sources	
Minerals	Eggshell	Seashell
Calcium	34.40 ± 2.37	38.04 ± 1.28
Phosphorus	0.27 ± 0.04	0.03 ± 0.02

**Note:**

Values are the mean percent composition of minerals in eggshells and seashells.

**Table 4:** Percent composition of calcium and phosphorus in the experimental diets.

	Experimental diets							
Minerals	0% ES	3% ES	6% ES	6% SS	9% ES	One way ANOVA		
						F	dF	*P*
Calcium	0.33 ± 0.01^a^	0.88 ± 0.01^b^	2.43 ± 0.04^ab^	2.85 ± 0.03^c^	4.36 ± 0.05^bc^	61,349.26	4.10	0.00
Phosphorus	1.27 ± 0.03^a^	1.30 ± 0.03^b^	1.36 ± 0.02^ab^	0.93 ± 0.02^c^	1.43 ± 0.03^bc^	1,121.10	4.10	0.00
Ca/P Ratio	0.26 ± 0.02^a^	0.68 ± 0.01^b^	1.78 ± 0.03^ab^	3.06 ± 0.02^c^	3.04 ± 0.03^c^	29,142.75	4.10	0.00

**Notes:**

Values are the mean percent composition of minerals in experiment diets.

Different superscripted letters in each column indicate significant differences (*P* < 0.05).

#### Growth performance

The average final wet weight of the freshwater crayfish fed experimental diets in individual cultures was between 0.76 ± 0.02 to 0.86 ± 0.02 g. There was a significant difference observed in the percentage body weight gain for individual culture experiments (one-way ANOVA, F = 9.51; dF = 4.10; *P* = 0.00). The average body weight gain of crayfish fed with 6% ES was found to be the highest among all the treatments. There was no significant difference in body weight gain between diet with eggshells and a diet with seashells as calcium sources (*P* > 0.05) (Table 5).

**Table 5:** Mean and standard deviation (± SD) of the growth performance and feed efficiency of juvenile red claw crayfish *Cherax quadricarinatus* fed formulated diet after 60 days.

	Experimental diets^1^							
Growth Performance	0% ES	3% ES	6% ES	6% SS	9% ES	One way ANOVA		
						F	dF	*P*
Initial weight (g)	0.20 ± 0.01^a^	0.21 ± 0.01^a^	0.20 ± 0.01^a^	0.21 ± 0.01^a^	0.21 ± 0.01^a^	0.55	4.10	0.74
Final weight (g)	0.76 ± 0.02^a^	0.81 ± 0.02^a^^b^^c^	0.86 ± 0.02^c^	0.86 ± 0.04^c^	0.83 ± 0.03^b^^c^	11.65	4.10	0.00
Total feed intake (g)	1.10 ± 0.02^a^	1.10 ± 0.01^a^	1.11 ± 0.01^a^	1.12 ± 0.04^a^	1.12 ± 0.01^a^	0.40	4.10	0.84
Weight gain (%)	277.22 ± 11.82^a^	282.27 ± 22.09^a^^b^	324.68 ± 5.48^c^	309.67 ± 5.14^b^^c^	293.86 ± 6.92^a^^b^^c^	9.51	4.10	0.00
SGR (%/day)	2.21 ± 0.05^a^	2.23 ± 0.09^a^^b^	2.41 ± 0.02^c^	2.35 ± 0.02^b^^c^	2.28 ± 0.03^a^^b^^c^	8.88	4.10	0.00
FCR	1.96 ± 0.04^b^^c^	1.82 ± 0.01^a^^b^^c^	1.68 ± 0.02^a^	1.72 ± 0.11^a^	1.81 ± 0.03^a^^b^	13.72	4.10	0.00

**Notes:**

Different superscripted letters in each column indicate significant differences (*P* < 0.05).

1 Refer to Table 1 for diet designations.

The highest SGR was obtained in juvenile crayfish fed with 6% ES (2.41%/d), followed by 6% SS (2.35%/d), 9% ES (2.28%/d), 3% ES (2.23%/d), and 0% ES (2.21%/d). There was a significant difference in the overall SGR among dietary treatments (one-way ANOVA, F = 8.88; dF = 4.10; *P* = 0.00). There was also no significant difference between eggshell and seashell calcium sources used in supplementing the diet (*P* > 0.05). There was a significant difference in FCR values for all the dietary treatments (one-way ANOVA, F = 13.72; dF = 4.10; *P* = 0.00). Meanwhile, the FCR of 6% SS (1.72) was not significantly different from 6% ES (*P* > 0.05) (Table 5).

**Figure 1:**
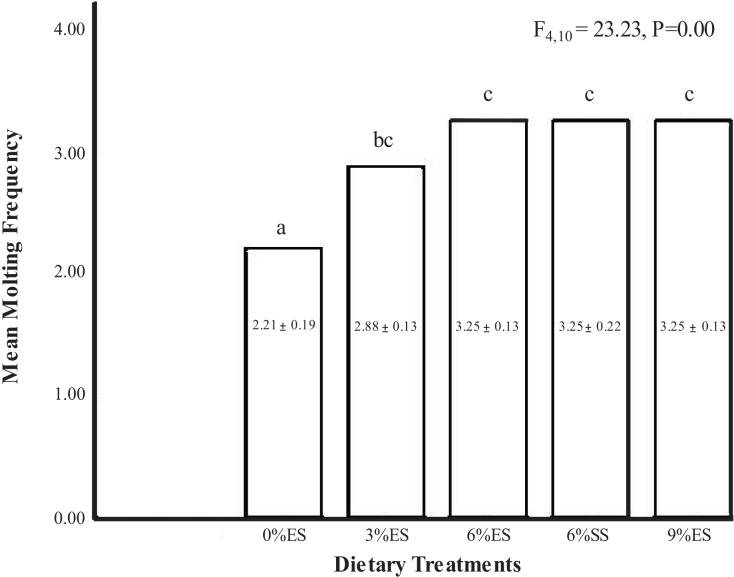
Mean molting frequency of *C. quadricarinatus* fed with different dietary treatments.

The highest molting frequency was observed to be the same (3.25 ± 0.22) and (3.25 ± 0.13) in juvenile crayfish fed with 6% SS and 6% ES, followed by 9% ES (3.25 ± 0.13), 3% ES (2.88 ± 0.13) and 0% ES (2.21± 0.19). In general, the dietary treatments significantly influenced the molting frequency (one-way ANOVA, F = 23.23; dF = 4.10; *P* = 0.00). However, diets 6%ES, 6%SS, and 9%ES were not significantly different from each other (*P* > 0.05) (Fig. 1).

**Figure 2:**
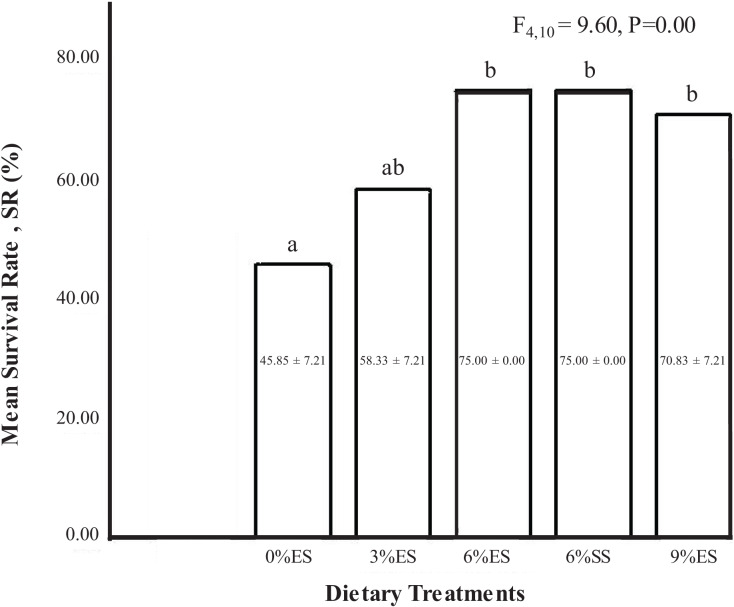
Mean survival rate of *C. quadricarinatus* fed with different dietary treatments.

The survival rate of juvenile crayfish ranged from 45% to 75%. The highest survival rate was recorded in crayfish fed with an experimental diet supplemented with 6% eggshell. On the other hand, the lowest survival rate was observed in the crayfish group fed with 0% calcium. There was a significant difference in survival rate between overall dietary treatments (one-way ANOVA, F = 9.60; dF = 4.10; *P* = 0.00). However, the survival rate of crayfish in 6% ES, 6% SS, and 9% ES was not significantly different from each other (*P* > 0.05) (Fig. 2).

**Figure 3:**
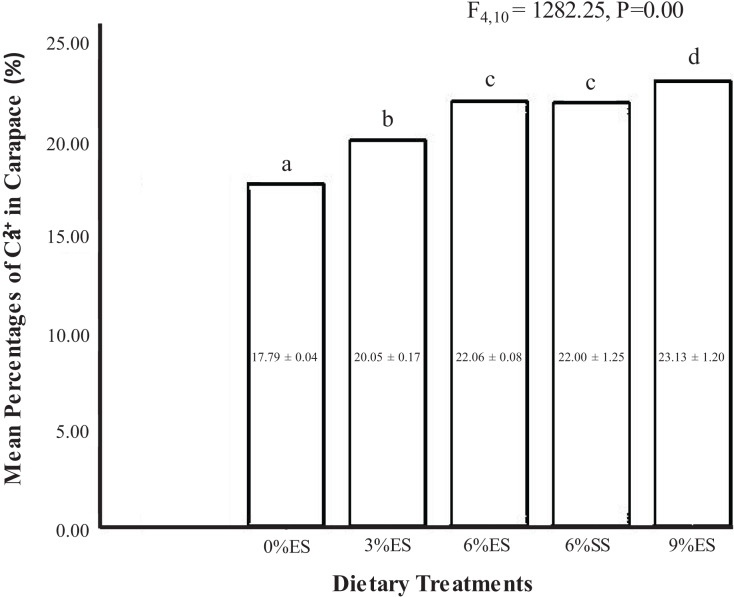
Mean calcium percentages in the carapace of *C. quadricarinatus* fed with different dietary treatments.

The highest calcium (Ca²^+^) content was found in juvenile crayfish fed with a 9% ES diet (23.1%) and the lowest was found in juvenile crayfish fed with a 0% calcium-supplemented experimental diet (17.8%). There was a significant difference in the percentage of calcium in the carapace for overall dietary treatments (one-way ANOVA, F = 1,282.25; dF = 4.10; *P* = 0.00). However, there was no significant difference in calcium content found in the crayfish exoskeleton between crayfish fed with eggshell and seashell-supplemented experimental diets (*P* > 0.05) (Fig. 3).

### Correlation between growth performance

**Figure 4:**
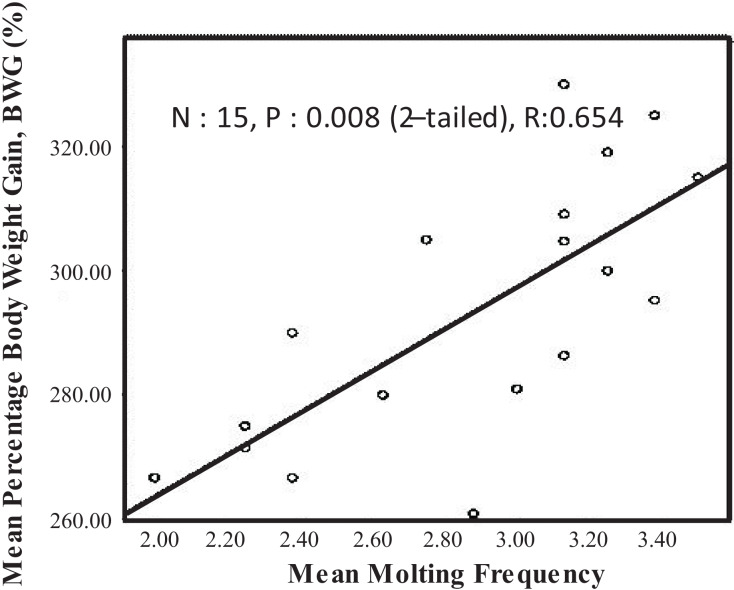
Pearson correlation, R-Test between mean molting frequency and mean percentage body weight gain, BWG (%) of C. quadricarinatus.

The results of the present study show that as the molting frequency increased, the body weight gain of juvenile crayfish also increased. The molting frequency was strongly related to the body weight gain of juvenile crayfish (N:15, P:0.008, R:0.654) (Fig. 4).

**Figure 5:**
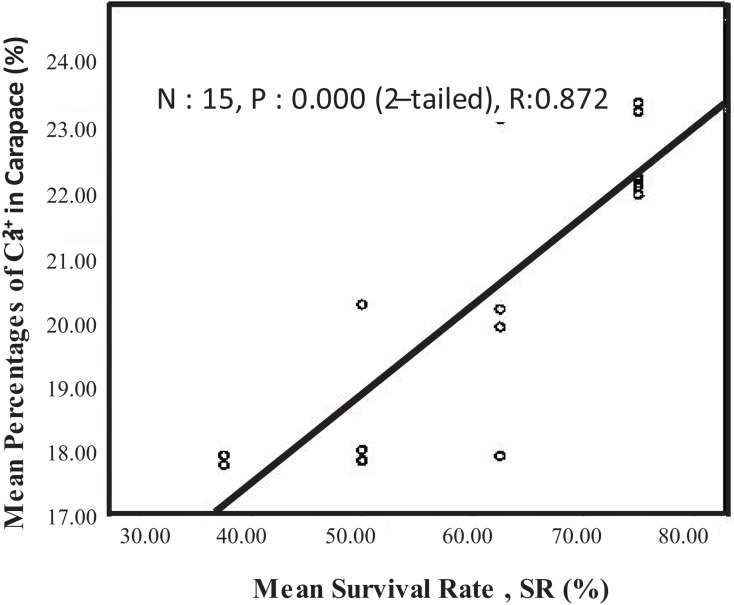
Pearson correlation, R-test between mean survival rate, SR (%) and mean percentages of Ca^2+^ in carapace (%) of *C. quadricarinatus*.

When the calcium content in the carapace increased, the survival rate also increased. The calcium content in the carapace was also strongly related to the survival rate of juvenile crayfish (N:15, P:0.000, R:0.872) (Fig. 5).

## Discussions

Fast growth and survival rate are important considerations in successful crayfish production. There are several factors that affect crayfish growth such as type of food, dietary supplementation, age, season, and stocking density (Lowery, 1988; Paglianti & Gherardi, 2004). Calcium is one of the most essential minerals for crustacean’s growth and physiological activities (Hessen, Kristiansen & Lid, 1991; Lovell, 1989). In the present study, the addition of calcium carbonate in crayfish diets resulted in a positive effect on body weight gain and SGR. Better body weight gain was observed when crayfish were fed with 6% natural calcium-supplemented diets compared to other inclusion levels. A similar study on freshwater crayfish juvenile, *Pontastacus leptodactylus*, showed that the best growth occurred when they were fed with a 6% calcium carbonate-supplemented diet (Mazlum & Şİrİn, 2020). Past studies also indicated that the growth of catfish (*Ictalurus punctatus*), rainbow trout (*Salmo gairdnerii*), and carp (*Cyprinus carpio*) were improved with feeding them diets supplemented with calcium (Andrews, Murai & Campel, 1973; Robinson et al., 1986). In the absence of calcium, molting activity progresses slower. Hence, the soft-shell period lasts longer and during this period, crayfish became weak which result in slower eating behavior compared to the normal phase (Stein, 1977). This explains why better growth is observed when crayfish are fed calcium-supplemented diets. The difference in growth at different calcium levels may also be due to the changes in crayfish metabolic activity (Sirin & Mazlum, 2017).

Dietary phosphorus also plays a key role in cellular energetics and is a constituent of cell membranes, nucleic acids, and skeleton (Roy & Lall, 2003; NRC, 2011). Several investigations on aquatic animals have suggested complex and interconnected roles of phosphorus in regulating their physiological functioning and healthy survival (Tewari et al., 2004). The nutritional needs of phosphorus in the crayfish should be comprehensively investigated together with the phosphorus level in the culture medium, its uptake efficiency, and other aspects that influence phosphorus metabolism. Studies carried out on crustaceans have indicated the importance of ratios between calcium and phosphorus in the growth of carapace and molting frequency. In the present study, diet 6% ES having Ca/P ratio of 1.78 yielded the best overall growth performance, but the value was not significantly different from diets 6% SS and 9% ES which contained similar Ca/P ratios of 3.06 and 3.04, respectively. There is a considerable range in the ratios in different crustaceans. In freshwater crayfish, *Astacus leptodactylus* the recommended Ca/P ratio has been reported to be 3.0 (Zahmetkesh et al., 2009), 1.01 in juvenile lobster, *Homarus americanus* (Gallagher et al., 1977); 1.0 in *Penaeus japonicus* (Kitabayashi et al., 1971) and 2.4–3.5 in rice-field crab (Panalikul, Wongmaneeprateep & Doolgindachbaporn, 2017; Nonpila, 2007). The inconstancy in the ratio was attributed to differences in target species, feed ingredients and their nutritional quality, chemical condition of the water and the experimental design of the culture system (Hassaan et al., 2013). In general, a Ca/P ratio of less than two is recommended when formulating commercial feeds for crustaceans for good growth performance (Davis & Gatlin, 1996).

Calcium content in diets from organic calcium sources has been observed to be sufficient to support growth in fish (Hassaan et al., 2013) but for crustaceans, there is a need to supplement dietary calcium with uptake from seawater to fulfill their metabolic requirements ostensibly due to repeated molting (Laining et al., 2011). There is no data on crayfish, but because molting follows much the same pattern in crustaceans, this species can probably modulate its uptake of calcium and phosphorus depending on intake through feed within a certain range. Excess calcium supply relative to phosphorus can adversely affect growth and survival (Davis, Lawrence & Gatlin, 1993). Crayfishes, like most crustaceans, are known for calcium carbonate mineralization of most of their skeleton but published information also suggests the significance of phosphate.

During the experiment, the water parameters were within the optimum range. At the end of the feeding trial, different levels of calcium significantly influenced the survival of red claw crayfish. Similar to growth performance, the highest survival rate was also observed when crayfish were fed a 6% eggshell-supplemented diet. The survival rate increased with the increased amount of calcium in the diets. In a previous study by Taugbol, Skurdal & Fjeld (1996), survival of crayfish was proportional to an increase in calcium level in diet and water, whereas the lowest survival rate was seen in crayfish fed with a 2% calcium-supplemented diet (Zahmetkesh et al., 2007). The essential need for this mineral is mainly because of the deposition of calcium carbonate (CaCO_3_) in the new exoskeleton in the calcifying process that causes its hardening in the post-molt and maintenance. Observations on growth and survival leave no doubt about the effect of calcium on the crayfish and the regulatory mechanism that is required to conserve and mobilize it for biological needs and metabolic homeostasis. Such a system is necessary since calcium requirements vary according to the stage of life, especially in the transition from intermolt (zero net flux) to premolt (net flux) and postmolt (net flux) (Zanotto & Wheatly, 2002). With the physiological mechanism governing molting essentially similar in crustaceans, the findings on crabs by Zanotto, Pinheiro & Sa (2009) on calcium balance in terms of ingestion as well as excretion through gills to achieve a balanced stage in the body could explain the physiological dynamics in the crayfish.

Eggshells and seashells are two sources of calcium carbonate that are abundantly available. Both have very limited use and are usually disposed of as waste products. The source of calcium (eggshell and seashell) had no significant effect on crayfish growth performance due to their similarity in chemical composition, especially calcium content. It was also noted that the addition of eggshells or seashells had no significant effect on the water hardness. This is because calcium carbonate (CaCO₃) has very low solubility in water. In aragonite and calcite forms, the solubility of CaCO₃ in water is known to be 16.6 mg/L at 20 °C water temperature. While in vaterite form, the solubility reduces to 6.6 mg/L at 20 °C water temperature. The solubility of CaCO₃ in water can be increased by adding ammonium salt or carbon dioxide (CO₂) into the water (Omari et al., 2016). A high level of water calcium hardness was reported to lower the survival rate in freshwater prawns, *Macrobrachium rosenbergii* (Adhikari et al., 2007). Hence, since CaCO₃ is insoluble in water, the addition of excess CaCO₃ in the feed or water system will not result in any significant changes in calcium water hardness which in fact might lead to stress to crayfish.

In the present study, both eggshell and seashell at 6% inclusion in the diets produced crayfish with a better molting frequency than other inclusion levels. Sirin & Mazlum (2017) reported that crayfish fed with a diet supplemented with 6% calcium chloride molted more frequently (MF:80.6%) compared to 3% (MF:49%) and 12% (MF:43.3%). The highest molting frequency had the highest survival rate in the present study. In the lowest molting frequency group, mortality was due to the incomplete molting process rather than cannibalism. The use of sufficient shelter in the form of polyvinyl chloride (PVC) pipes in the present study successfully provided the crayfish with a hiding area. This finding is aligned with Mason (1979) finding that the survival of freshwater crayfish, *Pacifastacus leniusculus* is enhanced with the addition of shelters in tanks. The presence of shelter also reduces the probability of injuries for freshwater crayfish (Savolainen, Ruohonen & Tulonen, 2003). Based on the Pearson correlation analysis between molting frequency and body weight gain, it was observed that molting frequency increased proportionally with body weight gain and both data were strongly related to each other (R:0.654).

The presence of calcium (Ca^2+^) in the carapace acts as an indicator of carapace quality. In the present study, we found that the percentage of calcium in carapace increased with an increase in calcium inclusion in the diets. However, there was no significant difference in calcium percentage in the carapace of crayfish-fed eggshell and seashell-supplemented diets. According to France (1987), the range of Ca^2+^ content in freshwater crayfish, *Orconectes virilis* carapace collected from their natural habitat is between 20–25%. The finding is aligned with our research findings. We found that the range of Ca^2+^ content in freshwater crayfish, *Cherax quadricarinatus* carapace fed with diets containing calcium carbonate (3–9%) is between 20–23%. This finding shows that providing a suitable diet for indoor crayfish farming that results in the same carapace quality in natural habitat is essential for optimal crayfish growth. The calcium content in the carapace was strongly related to the survival rate of juvenile crayfish (Pearson correlation, R: 0.872). Generally, calcium accumulates in the outer crust of many crustaceans including freshwater crayfish (Rukke, 2002). In a study by Mazlum & Şİrİn (2020), gastrolith accumulation was used to evaluate the level of calcium deposits in the carapace and found crayfish fed with the highest inclusion at 12% had the highest calcium level. Sufficient amount of calcium in gastrolith will ensure successful molting and faster shell hardening (Taugbol, Skurdal & Fjeld, 1996). Reduction of survival rate due to insufficient amount of dietary calcium was reported for the American lobster, *H. americanus* (Gallagher et al., 1978), and *P. japonicas* (Kanazawa, Teshima & Sasaki, 1984). It was observed that the protein, fat, moisture, and ash contents of the crayfish fed by adding different levels of calcium carbonate to the feed were not different among the treatment groups for *A. leptodactylus* (Mazlum & Şİrİn, 2020). However, there were significant differences in lipid and ash contents inside the tail meat for *A. leptodactylus* fed with another type of calcium supplement in the form of calcium chloride (CaCl₂) (Sirin & Mazlum, 2017).

Eggshells are generally favored as compared to seashells as a calcium source even though both have the same chemical composition. This is because eggshell supply is more sustainable and readily available in Malaysia while seashell sustainability is controversial due to the fact that seashell is part of body animal that has died and is usually found on the beach (Sali, 2015).

## Conclusion

Supplementation of eggshells and seashells significantly improved growth rate, FCR, molting frequency, and percentage of calcium in the exoskeleton. However, there was no significant difference in overall performance between eggshells and seashells as calcium sources in the diets for crayfish. The inclusion of 6% eggshell is recommended for the crayfish diet for better growth performance considering its low cost, abundance, and ease of processing. The use of natural calcium especially from eggshells will be able to reduce feed costs and support the sustainability of aquaculture production. It can play an important role in introducing a key circular economy perspective in aquaculture. Further investigations on calcium uptake and bioavailability in red claw crayfish are required for better insights into the performance of dietary calcium in the dynamics of metabolism. Such data on the crayfish should be of some relevance to other farmed crustaceans.

## Supplemental Information

10.7717/peerj.15449/supp-1 Supplemental Information 1 Raw data for Table 2.

10.7717/peerj.15449/supp-2 Supplemental Information 2 Raw data for Table 3.

10.7717/peerj.15449/supp-3 Supplemental Information 3 Raw data for Table 4.

10.7717/peerj.15449/supp-4 Supplemental Information 4 Raw data for Table 5.

10.7717/peerj.15449/supp-5 Supplemental Information 5 Raw data for Figures 4 and 5.

## Additional Information and Declarations

### Data Availability

The following information was supplied regarding data availability: The raw data are available int the Supplemental Files.
